# A Scientific Perspective of Personalised Gene-Based Dietary Recommendations for Weight Management

**DOI:** 10.3390/nu11030617

**Published:** 2019-03-14

**Authors:** Theresa Drabsch, Christina Holzapfel

**Affiliations:** Institute for Nutritional Medicine, University Hospital Klinikum rechts der Isar, Technical University of Munich, Georg-Brauchle-Ring 62, 80992 Munich, Germany; theresa.drabsch@tum.de

**Keywords:** gene-based, personalised nutrition, dietary recommendation, nutrigenetics, direct-to-consumer test, genotype, gene–diet interaction, weight loss, obesity

## Abstract

Various studies showed that a “one size fits all” dietary recommendation for weight management is questionable. For this reason, the focus increasingly falls on personalised nutrition. Although there is no precise and uniform definition of personalised nutrition, the inclusion of genetic variants for personalised dietary recommendations is more and more favoured, whereas scientific evidence for gene-based dietary recommendations is rather limited. The purpose of this article is to provide a science-based viewpoint on gene-based personalised nutrition and weight management. Most of the studies showed no clinical evidence for gene-based personalised nutrition. The Food4Me study, e.g., investigated four different groups of personalised dietary recommendations based on dietary guidelines, and physiological, clinical, or genetic parameters, and resulted in no difference in weight loss between the levels of personalisation. Furthermore, genetic direct-to-consumer (DTC) tests are widely spread by companies. Scientific organisations clearly point out that, to date, genetic DTC tests are without scientific evidence. To date, gene-based personalised nutrition is not yet applicable for the treatment of obesity. Nevertheless, personalised dietary recommendations on the genetic landscape of a person are an innovative and promising approach for the prevention and treatment of obesity. In the future, human intervention studies are necessary to prove the clinical evidence of gene-based dietary recommendations.

## 1. Body Weight Regulation

The regulation of body weight is of a complex nature. In addition to energy intake and expenditure, physiological parameters, feedback, and interaction systems of hormones, as well as the central nervous system, play a major role in body weight regulation. Signals of hunger and satiety are transmitted from fat tissue, muscles, and the gastrointestinal tract to brain areas. One of the satiety hormones is leptin, which is released by the adipose tissue and regulates the neuropeptide expression in the hypothalamus [1,2]. Leptin deficiency leads to extreme obesity and presents the most popular form of monogenic obesity [3]. Another hormone of hunger and satiety is ghrelin, which is secreted in the gastrointestinal tract after energy intake, and which is involved in glucose, lipid, and energy metabolism [4]. There are many other hormones which are involved in the regulation of hunger and satiety such as insulin, cholecystokinin, or glucagon-like peptide 1 [1,5,6]. For body weight maintenance, a balanced energy homeostasis is necessary. Therefore, lifestyle factors leading to a positive or negative energy balance result in weight gain or loss, respectively.

## 2. Dietary Intervention and Weight Loss

Lifestyle changes based on increasing physical activity and reducing energy intake are the basic therapeutic approaches for weight loss and weight maintenance. It is well known that not the macronutrient composition of a diet, but the energy content resulting in a negative energy balance plays the major role for weight loss. This guarantees a higher flexibility for experts and patients in the choice of a dietary concept in order to reach a hypocaloric diet for weight loss. Additionally, food preferences and wishes of patients, as well as the suitability of the dietary strategy for the daily routine can be taken into account. An evaluation of 48 studies showed that, regardless of the macronutrient composition of a diet, the extent of weight loss was similar within six and 12 months [7]. In another study, four diets consisting of different macronutrient compositions were investigated. After two years, there was no significant difference in weight loss between the four intervention groups [8]. Another study by Shai et al. showed that weight reduction after two years was independent of the macronutrient composition of the diet [9]. This effect was confirmed in a study on 609 adults with a body mass index (BMI) between 28 and 40 kg/m^2^ [10]. Gardner et al. found out that, after one year of dietary intervention, mean weight loss was not significantly different as individuals lost 6.0 kg of weight in the low-carbohydrate diet group and 5.3 kg of weight in the low-fat diet group [10]. A meta-analysis of 16 randomised controlled trials including 3436 individuals suggests that a Mediterranean diet leads to a significantly higher weight loss compared to a control diet (mean difference between diet groups: −1.75 kg), especially if diet was energy-reduced and was associated with an increased physical activity [11]. Based on this finding and further studies, it might be concluded that alternative diets such as the plant-based form of the Atkins diet or the Mediterranean diet may lead to a moderate weight loss [9,12,13]. However, due to the saturation effect of protein-rich diets, an increased protein intake is also often aimed at in nutritional weight loss concepts. Another aspect is the quality of fats. Studies showed that an increased consumption of omega-3 or omega-6 polyunsaturated fatty acids improves plasma lipid levels as well as the risk for cardiovascular events [14,15]. In addition, new information and communication technologies such as mobile applications are popular for making self-help recommendations for weight loss [16]. Due to eating preferences, as well as individual metabolic responses on dietary intake and large variations in weight loss success, the need of an individual nutritional recommendation instead of a “one size fits all” is increasing. Personalised dietary recommendations are of high potential for an improved and more successful weight management. However, the nature and the extent of personalised dietary recommendations are still unknown.

## 3. Individual Metabolic Response to Dietary Intervention

In the last few years, studies showed that persons individually respond to predefined meal challenges. In the Human Metabolome (HuMet) study, 15 males were investigated for metabolic responses to specific challenges [17]. After a fasting period of 36 h, participants underwent an oral glucose and lipid test, liquid test meals, and exercises, and they were exposed to cold. Due to deep phenotyping and the healthy nature of the participants, Krug et al. could show large variability in metabolic responses between phenotypically similar individuals after challenges by test meals or exercise programmes [17]. Another study investigated the metabolic response to identical meals in 800 participants. In this Israeli study, blood glucose levels of the participants, aged 18–70 years, were analysed during a standardised meal resulting likewise in a large inter-individual variability [18]. The average postprandial glycaemic response (PPGR) differed largely between individuals (e.g., bread: 44 ± 31 mg/dL·h (mean ± standard deviation). This inter-individual difference of glycaemic response validated the fact that the same meal may lead to another or even the opposite metabolic response when comparing different individuals. In a sub-study, participants were assigned to a predicted “good” or “bad” diet based on the glucose levels. The results showed that personalised dietary interventions can lead to improved PPGR [18]. Another study by the same research group analysed the individual PPGR to different types of bread [19]. In that randomised cross-over trial, participants received a white or sourdough-leavened bread. A large inter-individual variability in PPGR to the two kinds of bread was confirmed. Some subjects had a higher glycaemic response to one bread and some to the other [19]. In a cross-over study, the metabolic response of 20 healthy male volunteers and 20 male patients with type 2 diabetes to a PhenFlex test drink or glucose drink (OGTT) was investigated [20]. The PhenFlex test used a drink consisting of 60 g of fat, 75 g of glucose, and 20 g of protein. OGTT corresponds to the commonly used drink with 75 g of glucose. A total of 132 metabolic parameters were quantified as markers for 26 different metabolic processes. The results showed a significant difference between the two groups, indicating different phenotypic flexibility, especially in metabolically impaired individuals [20]. The explanations for the differences in metabolic response are complex and widely discussed. Genetic parameters, as well as the microbiome, might play a role and are of high potential to explain a certain amount of the inter-individual metabolic differences upon meal challenges.

## 4. Purpose of This Work

The purpose of this article was to provide a science-based viewpoint on gene-based personalised nutrition and weight management. This means that the scientific background of the commercially available direct-to-consumer (DTC) genetic tests was questioned. Furthermore, human intervention studies investigating the effect of gene-based dietary recommendations on weight change are described in order to present the ongoing research. This viewpoint combines different perspectives (science, clinical evidence, practical issues) and various aspects (scientific results, commercially available offers) and discusses recent issues aiming to highlight the current evidence of gene-based personalised nutrition.

## 5. Definition of a Gene-Based Personalised Diet

**Figure 1:**
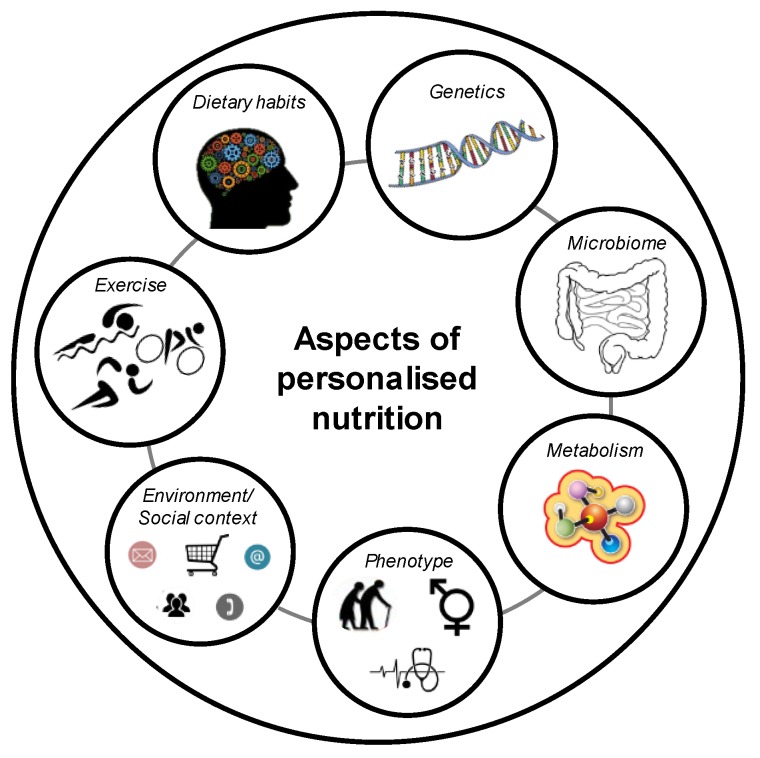
Aspects of personalised nutrition.

To date, there is no single definition of a personalised diet. Personalised nutrition is also called precision or tailored nutrition [21]. Nizel et al. defined personalised nutrition with a personal consultation of patients in order to achieve an improvement in dietary habits [22]. Subsequently, further tools were included, such as online available platforms or applications based on dietary and behavioural habits of each patient as a kind of computer-generated personalised nutrition [23]. Another concept defined as “personalised, gene-based nutrition” combines genetic information with specific dietary recommendations. In 2013, Stewart-Knox et al. described a personalised nutrition as a healthy dietary recommendation tailored to the health status, lifestyle, and/or the genetic information of an individual [24]. Lifestyle data included age, gender, height, weight, and clinical facts such as disease history, food allergies, or intolerances, as well as dietary habits and exercise behaviour. Wang and Hu included at least the microbial composition to improve dietary recommendations [25]. Furthermore, personalised nutrition is directly related to nutrigenetics [26]. However, direct translation from a genetic profile to the phenotypic characterisation of a person is of a complex nature. Therefore, the concept of personalised dietary recommendations has to follow a multi-dimensional approach considering, e.g., social, lifestyle, genetic, and metabolic parameters. Different aspects of a personalised nutrition are described in Figure 1.

The major aim of a personalised nutrition, according to Daniel and Klein, should be a dietary recommendation adjusted to an individual’s requirements by including, if necessary, dietary recommendations based on phenotype and genotype to maintain the health status and to counteract risks for diseases or their comorbidities [27]. In a double-blinded randomised controlled trial, short- and long-term effects on dietary intake of a gene-based personalised nutrition were investigated [28]. In this study, Nielsen and El-Sohemy showed that there was no significant difference in dietary intake after three months of intervention between the intervention group receiving information on their genetic background and, additionally, a corresponding gene-based dietary recommendation and the control group. After 12 months, some significant improvements in dietary intake such as a reduced intake of sodium in the personalised nutrition group were observed, suggesting a long-term change in dietary habits [28]. Nevertheless, the exact mechanisms and factors influencing the long-term effect of a personalised nutrition are still unclear.

Another aspect is the psychological effect of a personalised dietary recommendation. In a survey, 9381 participants from nine European countries were interviewed [29]. The questionnaire was based on results of an explorative analysis and data from the literature. The study showed that the greater the participant’s benefit of a personalised dietary recommendation is, the more positive the respective attitudes are and the greater the probability that such a recommendation will be accepted [29]. The results of this study also indicate that the provider’s presentation of the potential benefits, the efficacy of regulatory control, and the protection of consumers’ personal data are major concerns for the adoption of personalised dietary recommendations. These aspects are also in line with the Health Belief Model [30]. This model describes that changes in health behaviour are more likely if the associated benefits are experienced as high, while individual burdens (“costs”) are perceived as low. Another point, suggested by Anderson, is the consideration of the social environment for personalised dietary recommendations to maximise the individual success and the change to healthy eating behaviour [31]. The social network, e.g., contact with a partner or a group, could prevent unhealthy eating behaviour through regular contact, monitoring of each other’s weight change, and solving problems together.

## 6. Genetics and Obesity

The first studies investigating the association between a genetic background and body weight were focused on the heritability of body weight by analysing twins or adopted children. Bouchard et al. could show that, after overfeeding, the differences in increasing body weight were higher between twin pairs than within one twin pair [32]. In adoption studies, the BMI of adopted children was more associated with the BMI of their biological parents than with the BMI of their non-biological parents [33]. However, in hypothesis-driven candidate gene studies, a significant association between genetic loci and body weight was identified. The investigated genes were mainly chosen due to biological plausibility and had a function in regulating food intake, played a role in lipid metabolism, or were involved in the excretion of intestinal hormones. For instance, the fatty-acid-binding protein 2 (*FABP2*) is expressed by epithelial cells of the small intestine where it is mainly related to fat absorption. Variants in the *FABP2* genetic locus lead to increased fat absorption and are associated with obesity [34,35]. Another example is the peroxisome proliferator-activated receptor-gamma (*PPARG*) gene, which is expressed in fat cells and, thus, plays a key role in the differentiation of adipocytes [36,37]. Deeb et al. could show that the *PPARG* gene is associated with BMI and insulin sensitivity [38]. In hypothesis-free, genome-wide association studies (GWAS), many genetic loci were identified for an association with body weight [39,40,41,42]. However, only 2.7% of the variation in BMI might be explained by these genetic loci [40]. Up to now, around 500 genetic loci are described for associations with adiposity traits such as BMI or waist-to-hip ratio [43]. To date, the fat mass and obesity associated (*FTO*) locus is the gene with the strongest effect on body weight. Frayling et al. could show that carriers of two risk alleles of the single nucleotide polymorphism (SNP) rs9939609 at the *FTO* locus weighed up to three kilograms more than the non-risk allele carriers [44]. This finding was confirmed by Dina et al. in French individuals [45] but not in African Americans [46]. Scuteri et al. explained this non-significant finding in African Americans by the ethnic-based differences of the genetic architecture of obesity. The *FTO* SNP rs9939609 might be quite common in Europeans but rare in African Americans [46]. Claussnitzer et al. showed that the *FTO* SNP rs1421085 influences the expression of two proxies of the iroquois homeobox family, resulting in the promotion of the expression of energy-storing white adipocytes and in the inhibition of energy-burning beige adipocytes [47]. Therefore, weight gain may not necessarily be the result of higher energy intake, but may be related to a reduced proportion of energy-burning adipocytes [47]. In addition to the *FTO* locus, further genetic variants at different loci were shown to be associated with body weight. One of these is the transmembrane protein 18 (*TMEM18*) gene. *TMEM18* is expressed throughout the body and plays a role in the regulation of body weight, appetite, and even in the development of obesity [48,49]. Another gene on chromosome 18 is the melanocortin-4 receptor (*MC4R*), whose risk allele is associated with 0.23 kg/m^2^ higher BMI [42]. This effect might be explained by the role of *MC4R* in the regulation of dietary intake [50]. Moreover, a deficiency of this gene leads to the most common monogenic form of obesity [51]. However, the biological function of most of the obesity-associated genetic loci remains unclear [43]. In the future, the identification of rare and causal genetic variants might serve for drug development for weight loss.

## 7. Genetics and Weight Loss

It seems plausible that obesity-associated genetic variants are also associated with weight loss. Therefore, studies investigated the association between SNPs and weight change. In a systematic review and meta-analysis, Xiang et al. meta-analysed 10 weight loss intervention studies [52]. In this meta-analysis, the *FTO* risk allele A carriers had significantly greater weight loss than non-risk allele carriers. However, in another systematic review and meta-analysis on the association between the *FTO* gene and weight loss, Livingstone et al. summarised the findings of eight randomised controlled trials including 9563 adults [53]. Results of that meta-analysis showed that people carrying the *FTO* risk allele of SNP rs9939609 achieved a similar weight loss compared to non-risk allele carriers after dietary intervention. Livingstone et al. justified the different outcomes of the two meta-analyses with the fact that, despite a small overlap of the included studies, the population size in the work of Livingstone et al. was considerably larger and only randomised trials were considered for inclusion [53]. This non-significant difference between risk and non-risk allele carriers is in line with an intervention study which investigated 26 obesity-related loci and their association with weight loss [54]. In this randomised controlled trial conducted in eight clinical centres in Europe, 771 adults with obesity underwent a 10-week dietary hypocaloric intervention. There were no significant differences in weight loss when risk allele carriers were compared to non-risk allele carriers [54]. Results of the recently published randomised controlled Diet Intervention Examining the Factors Interacting with Treatment Success (DIETFITS) study showed again that weight reduction of 609 adults with overweight was independent of genotypes [10]. In addition to the non-significant findings, a pooled analysis of studies showed a significant positive association between the risk G allele of the mitochondrial translational initiation factor 3 SNP rs1885988 and weight loss [55].

## 8. Genetics and Dietary Intake

The regulation of food intake, selection of macronutrients, and total energy intake is very complex. Some epidemiological and intervention studies investigated associations between genetic variants and dietary intake.

A systematic review and meta-analysis analysed data from epidemiological studies which investigated the association between the *FTO* genotype and macronutrient intake [56]. In this review, Livingstone et al. provided evidence for a significant association between carriers of *FTO* risk alleles and a reduced energy intake of around six kilocalories per day [56]. Another published systematic review provided an overview of a wide range of genetic loci and confirmed an inconsistency of findings concerning the relationship between genetic variants and energy intake [57]. A recently published GWAS investigated the relationship between genetic loci and energy intake in 18,773 individuals of European ancestry [58]. No significant association between genetic variants of the *FTO* gene and energy intake was found.

Livingstone et al. could further show a significant association between *FTO* risk allele carriers and an increased fat and protein intake [56]. In contrast to Livingstone et al. [56], the systematic review by Drabsch et al. [57] did not provide clear evidence of an association between *FTO* and carbohydrate or fat intake. This result was confirmed by Merino et al. who provided data of a large GWAS based on 91,114 individuals from 24 epidemiological studies [59]. No genome-wide significance for an association between the *FTO* SNP rs1421085 and carbohydrate or fat intake was shown. Only the association between the *FTO* SNP rs421085 and a higher protein intake was confirmed. In addition to the *FTO* genotype, further genetic loci were investigated for associations with macronutrient intake. Merino et al. identified two genetic loci, the retinoic acid receptor beta (*RARB*) locus and the deoxyribonucleic acid (DNA) damage regulated autophagy modulator 1 (*DRAM1*) locus, which showed genome-wide significance concerning a relation to macronutrient intake. The *RARB* SNP rs7619139 was positively associated with carbohydrate intake. Similarly, a significant association between rs77694286 at the *DRAM1* locus and a higher protein intake was shown. In addition to these findings, Merino et al. confirmed that the fibroblast growth factor 21 SNP rs838133 was associated with all macronutrient intakes [59].

In addition to epidemiological findings, results from intervention studies are of interest (Table 1). In the Nutrient–Gene Interactions in Human Obesity (NUGENOB) randomised trial, 771 adults with obesity were assigned to a 10-week dietary intervention based on two different hypocaloric diets [54]. A total of 42 SNPs at 26 genetic loci were examined. The results showed no significant interaction between genetic variants and dietary intervention on weight change [54]. This result was confirmed by the Diet, Obesity, and Genes (DiOGenes) study [60], in which 742 participants were randomly assigned to one of five diets based on different levels of glycaemic indices. However, findings could not provide significant evidence for 651 different SNPs and an interaction with diet on weight change [60]. In 2012, results of the randomised controlled trial Preventing Overweight Using Novel Dietary Strategies (POUNDS LOST) were published [61]. In this study, *FTO* risk allele carriers showed a significantly increased improvement in body weight change, body composition, and fat distribution compared to carriers of the non-risk allele. However, this effect was only observed if risk allele carriers of the *FTO* SNP rs1558902 followed a high-protein diet [61]. In a prospective analysis of this trial, Qi et al. described that homozygous risk allele C carriers of the insulin receptor substrate 1 SNP rs2943641 had higher weight loss than those with the non-risk genotype in the high-carbohydrate and low-fat diet group [62]. Furthermore, results of the Food4Me [63] and the DIETFITS [10] study were not significant (Table 1). In conclusion, none of the selected studies presented in Table 1 could show either a significant SNP-diet interaction on weight loss or a genotype-dependent effect on weight loss.

## 9. Direct-to-Consumer Tests

Gene-based dietary recommendations represent potential for commercial purposes. A number of companies already offer so-called DTC genetic tests (Table 2). The given dietary recommendation is based on the customer’s DNA sample. The genetic profile, which is determined by the commercial providers, is mainly based on gene variants, which were investigated in candidate gene studies and for which associations with metabolic functions or certain disease risks are known (Table 2). One example might be the *PPARG* locus, which is associated with insulin sensitivity and body weight [38].

**Figure 2:**
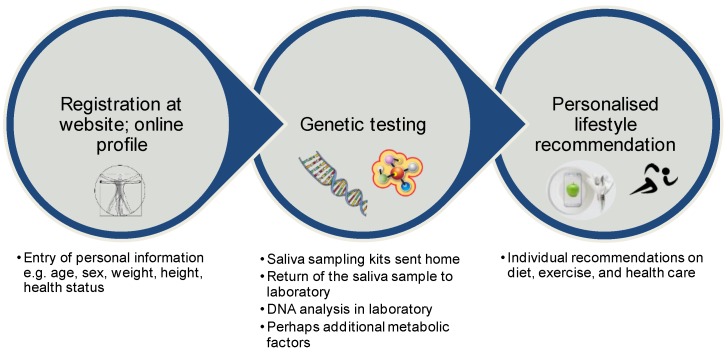
Schematic workflow of a commercially available gene-based dietary recommendation. DNA, deoxyribonucleic acid.

In general, a DTC test is defined as genetic test which is purchased directly by the consumer mostly via the internet [64]. In this case, genetic tests typically use saliva samples. Some companies only investigate one single genetic variant, while other DTC genetic test companies analyse several hundred SNPs [64]. The general pattern according to which these companies proceed is shown in Figure 2. After registration (online profile), the customer receives a box with tools, with which the genotype of each individual can be analysed using saliva samples. Based on the customer’s profile and the genetic background, the company provides a personalised dietary recommendation. Subsequently, customers receive their specific results of the genetic test, as well as the dietary recommendation by email or by downloading the material in the online account on the company’s website. In some cases, customers also receive a proposal for supplements that they can purchase directly from the company and that support the compliance of the gene-based dietary recommendations. The costs of a genetic DTC test vary largely between companies. In Table 2 some companies and their concepts are listed.

Studies investigated the effect of commercially available gene-based dietary recommendations on weight loss. In a prospective study sponsored by a company providing genetic DTC tests, 51 individuals with overweight or obesity were randomly assigned to a nutrigenetic guided diet or a standard control diet [65]. Focusing on the number of participants who lost 5% of body weight at eight or 24 weeks, there was no significant difference between the two diet groups [65]. This study was limited by a small sample size and a short duration. Another study using a commercially available genetic test also had several methodological limitations [66].

In addition to the genetic profile, some companies also include other aspects of human metabolism into their dietary recommendations. The company Habit (https://habit.com) investigates the metabolic response on carbohydrates, fats, and proteins (shakes) and includes this information into the personalised dietary recommendation. Another commercially available personalised dietary programme is provided by the Million Friends company (https://www.millionfriends.de), which includes continuous glucose monitoring and the analysis of the microbiome into the personalised dietary recommendations.

In a systematic review by Covolo et al., different aspects of DTC tests were summarised [67]. The clinical validity of the genetic tests and the benefits are still limited. Furthermore, a lack of scientific evidence is clearly pointed out. In addition, contradictions in the results of genetic tests on the same individuals were identified. Due to missing counselling, a high risk of misinterpretation of the genetic result is given. Covolo et al. concluded that the practical experiences are limited, and that this market is still in a premature state [67].

## 10. Current Opinions for Gene-Based Diets

As described above, there is little scientific evidence for genetic DTC tests. This is also reflected by the opinions of nutritional and genetic societies. The German Society for Human Genetics rejects the use of genetic tests in their positioning paper [68], and the American Society of Dietetics and Nutrition declined the use of gene-based dietary recommendations in clinical settings [69]. In their position paper they stated, “*The practical application of nutritional genomics for complex chronic disease is an emerging science and the use of nutrigenetic testing to provide dietary advice is not ready for routine dietetics practice. Registered dietitian nutritionists need basic competency in genetics as a foundation for understanding nutritional genomics; proficiency requires advanced knowledge and skills*”. A systematic review of 17 European position statements, policies, guidelines, and recommendations described the concerns of societies about the DTC tests referring to the quality, genetic understanding, and protection of privacy [70]. Despite the concerns of societies, the mentioned review points out that the concept should be strictly regulated and that a common European regulation on the use of genetic data is crucial [70]. In addition, medical staff should be given the best possible training in the field of genetic DTC tests. In addition to the opinions of public societies, even the personal opinions, wishes, and concerns of individuals should be taken into account [71]. Furthermore, ethnic-based genetic differences should be considered.

## 11. Outlook

Bray and colleagues clearly pointed out in their article that personalised dietary recommendations are a hot topic for future obesity therapy and that clinical studies are necessary [72]. The whole area of personalised nutrition is very complex, and it is of urgent need to focus on several aspects (Figure 1), and not only on the person´s genetic background. Therefore, it is indispensable to conduct multidisciplinary studies in order to bring all potential factors together for a valid personalised dietary recommendation. The topic of personalised nutrition was picked up in the framework of the *enable* cluster (http://www.enable-cluster.de), which is funded by the Federal Ministry of Education and Research in Germany. The aim of the lifestyle intervention (LION) study is to identify, e.g., genetic, epigenetic, metabolic, and psychological predictors and barriers for weight loss and weight loss maintenance.

## Figures and Tables

**Table 1:** Examples of studies investigating associations between genetic variants, dietary intake, and weight change.

Study	Investigated SNPs	Intervention	Result	Reference
NUGENOB	42 SNPs at 26 genetic loci	Ten-week dietary intervention based on two hypocaloric diets of 600 kcal/d each and percentage of energy derived from fat of 20–25% (low fat) or 40–45% (high fat)	No SNP–diet interaction on weight change	Sorensen et al. (2006) [54]
DiOGenes	651 SNPs at 69 genetic loci	Five different ad libitum diets consisting of different glycaemic indices (GI) and contents of dietary protein (P): low P/low GI vs. low P/high GI vs. high P/.ow GI vs. high P/high GI vs. control diet	No SNP–diet interaction on weight change	Larsen et al. (2012) [60]
Food4Me	5 SNPs at 5 genetic loci (*FTO*, *FADS1*, *TCF7L2*, *ApoE(e4*), *MTHFR*)	Four different diet groups: (1) Non-personalised dietary recommendation (2) Personalised dietary advice based on dietary habit (3) Personalised dietary advice based on dietary habit and phenotypic data (4) Personalised dietary advice based on dietary habit, phenotypic and genotypic data	No significant difference of weight change between risk and non-risk allele carriers; level of personal dietary advice had no effect on weight change	Celis-Morales et al. (2015) [63]
DIETFITS	3 SNPs at 3 genetic loci (*PPARG*, *ADRB2*, *FABP2*)	Low-fat diet or a low-carbohydrate diet	Similar weight change between groups independent of genetic pattern	Gardner et al. (2018) [10]

Examples for studies investigating associations between genetic variants, dietary intake and weight change. *ADRB2*, adrenoreceptor beta 2; *ApoE(e4)*, apolipoprotein E (e4); DIETFITS, the Diet Intervention Examining the Factors Interacting with Treatment Success randomised clinical trial; DiOGenes, the Diet, Obesity, and Genes study; *FABP2*, fatty-acid-binding protein 2; *FADS1*, fatty-acid desaturase 1; *FTO*, fat mass and obesity associated; *MTHFR*, methylenetetrahydrofolate reductase; NUGENOB, Nutrient–Gene Interactions in Human Obesity: Implications for Dietary Guidelines; *PPARG*, peroxisome proliferator-activated receptor-gamma; SNP, single nucleotide polymorphism; *TCF7L2*, transcription factor 7 like 2.

**Table 2:** Examples of companies offering gene-based dietary recommendations for weight loss.

Company	Genetic Approach	Dietary Recommendation Based on	Homepage
Pathway Genomics	SNPs at genetic loci such as *ADIPOQ* (rs17300539, rs17366568), *APOA2* (rs5082), *FADS1* (rs174547), *FTO* (rs9939609, rs1121980), *MC4R* (rs17782313), *PPARG* (rs1801282)	Genetic profile matched to a low-fat, low-carbohydrate, Mediterranean or balanced diet, including genetic risks for metabolic health factors (e.g., blood sugar, lipids)	https://www.pathway.com
Thinner Gene	SNPs at genetic loci such as *FTO*, *PPARG*, *PLIN*, *ADRB2*, *ADIPOQ*, *FABP2*, *PPARG*, *IRS1*, *APOA2/5*, *TCF7L2*	Genetic profile and sensitivity for carbohydrates, fats, and proteins matched with healthy food and fat control	http://www.thinnergene.com
Genetic Balance	SNPs at genetic loci associated with fat and carbohydrate metabolism	Genetic make-up matched to good or bad burning of carbohydrates or fats	https://www.genetic-balance.com
Bodykey by NUTRILITE	SNPs at genetic loci such as *FABP2* (rs1799883), *PPARG* (rs1801282), *ADRB2* (rs1042713), *ADRB2* (rs1042714), *ADRB3* (rs4994)	Genetic profile matched to diets with different macronutrient compositions	https://www.bodykey.at
Nutrigenes	100 SNPs at genetic loci such as *FADS1*	Genetic predisposition to food and nutrient needs, intolerances and sensitivities	http://www.nutrigenes.ch
My Kirée	Eight genetic loci associated with body weight	Genetic profile for fat or carbohydrate sensitivity, including supplementation with fat and carbohydrate blockers	https://my-kiree.com

All homepages were visited on 25th January 2019. *ADIPOQ*, adiponectin, C1Q, and collagen domain containing; *ADRB2/3*, adrenoceptor beta 2/3; *APOA2/5*, apolipoprotein A2/5; *FABP2*, fatty-acid-binding protein 2; *FADS1*, fatty-acid desaturase 1; *FTO*, fat mass and obesity associated; *IRS1*, insulin receptor substrate 1; *LIPC*, lipase C, hepatic type; *MC4R*, melanocortin 4 receptor; *PLIN*, perilipin 1; *PPARG*, peroxisome proliferator-activated receptor-gamma; *TCF7L2*, transcription factor 7 like 2.

## References

[1] Lam Y.Y., Ravussin E. (2016). Analysis of energy metabolism in humans: A review of methodologies. Mol. Metab..

[2] Crowley V.E. (2008). Overview of human obesity and central mechanisms regulating energy homeostasis. Ann Clin. Biochem..

[3] Montague C.T., Farooqi I.S., Whitehead J.P., Soos M.A., Rau H., Wareham N.J., Sewter C.P., Digby J.E., Mohammed S.N., Hurst J.A. (1997). Congenital leptin deficiency is associated with severe early-onset obesity in humans. Nature.

[4] Wiedmer P., Nogueiras R., Broglio F., D’Alessio D., Tschop M.H. (2007). Ghrelin, obesity and diabetes. Nat. Clin. Pract. Endocrinol. Metab..

[5] Woods S.C., D’Alessio D.A. (2008). Central control of body weight and appetite. J. Clin. Endocrinol. Metab..

[6] Raybould H.E. (2007). Mechanisms of CCK signaling from gut to brain. Curr. Opin. Pharmacol..

[7] Johnston B.C., Kanters S., Bandayrel K., Wu P., Naji F., Siemieniuk R.A., Ball G.D., Busse J.W., Thorlund K., Guyatt G. (2014). Comparison of weight loss among named diet programs in overweight and obese adults: A meta-analysis. JAMA.

[8] Sacks F.M., Bray G.A., Carey V.J., Smith S.R., Ryan D.H., Anton S.D., McManus K., Champagne C.M., Bishop L.M., Laranjo N. (2009). Comparison of weight-loss diets with different compositions of fat, protein, and carbohydrates. N. Engl. J. Med..

[9] Shai I., Schwarzfuchs D., Henkin Y., Shahar D.R., Witkow S., Greenberg I., Golan R., Fraser D., Bolotin A., Vardi H. (2008). Weight Loss with a Low-Carbohydrate, Mediterranean, or Low-Fat Diet. N. Engl. J. Med..

[10] Gardner C.D., Trepanowski J.F., Del Gobbo L.C., Hauser M.E., Rigdon J., Ioannidis J.P.A., Desai M., King A.C. (2018). Effect of Low-Fat vs Low-Carbohydrate Diet on 12-Month Weight Loss in Overweight Adults and the Association with Genotype Pattern or Insulin Secretion: The DIETFITS Randomized Clinical Trial. JAMA.

[11] Esposito K., Kastorini C.M., Panagiotakos D.B., Giugliano D. (2011). Mediterranean diet and weight loss: Meta-analysis of randomized controlled trials. Metab. Syndr. Relat. Disord..

[12] Jenkins D.J., Wong J.M., Kendall C.W., Esfahani A., Ng V.W., Leong T.C., Faulkner D.A., Vidgen E., Greaves K.A., Paul G. (2009). The effect of a plant-based low-carbohydrate (“Eco-Atkins”) diet on body weight and blood lipid concentrations in hyperlipidemic subjects. Arch. Intern. Med..

[13] Jenkins D.J., Wong J.M., Kendall C.W., Esfahani A., Ng V.W., Leong T.C., Faulkner D.A., Vidgen E., Paul G., Mukherjea R. (2014). Effect of a 6-month vegan low-carbohydrate (‘Eco-Atkins’) diet on cardiovascular risk factors and body weight in hyperlipidaemic adults: A randomised controlled trial. BMJ Open.

[14] Harris W.S., Mozaffarian D., Rimm E., Kris-Etherton P., Rudel L.L., Appel L.J., Engler M.B., Sacks F. (2009). Omega-6 fatty acids and risk for cardiovascular disease: A science advisory from the American Heart Association Nutrition Subcommittee of the Council on Nutrition, Physical Activity, and Metabolism; Council on Cardiovascular Nursing; and Council on Epidemiology and Prevention. Circulation.

[15] Bamberger C., Rossmeier A., Lechner K., Wu L., Waldmann E., Stark R.G., Altenhofer J., Henze K., Parhofer K.G. (2017). A Walnut-Enriched Diet Reduces Lipids in Healthy Caucasian Subjects, Independent of Recommended Macronutrient Replacement and Time Point of Consumption: A Prospective, Randomized, Controlled Trial. Nutrients.

[16] Holzmann S.L., Pröll K., Hauner H., Holzapfel C. (2017). Nutrition apps: Quality and limitations. An explorative investigation on the basis of selected apps. Ernaehrungs Umsch..

[17] Krug S., Kastenmuller G., Stuckler F., Rist M.J., Skurk T., Sailer M., Raffler J., Romisch-Margl W., Adamski J., Prehn C. (2012). The dynamic range of the human metabolome revealed by challenges. FASEB J. Off. Publ. Fed. Am. Soc. Exp. Biol..

[18] Zeevi D., Korem T., Zmora N., Israeli D., Rothschild D., Weinberger A., Ben-Yacov O., Lador D., Avnit-Sagi T., Lotan-Pompan M. (2015). Personalized Nutrition by Prediction of Glycemic Responses. Cell.

[19] Korem T., Zeevi D., Zmora N., Weissbrod O., Bar N., Lotan-Pompan M., Avnit-Sagi T., Kosower N., Malka G., Rein M. (2017). Bread Affects Clinical Parameters and Induces Gut Microbiome-Associated Personal Glycemic Responses. Cell Metab..

[20] Wopereis S., Stroeve J.H.M., Stafleu A., Bakker G.C.M., Burggraaf J., van Erk M.J., Pellis L., Boessen R., Kardinaal A.A.F., van Ommen B. (2017). Multi-parameter comparison of a standardized mixed meal tolerance test in healthy and type 2 diabetic subjects: The PhenFlex challenge. Genes Nutr..

[21] De Toro-Martin J., Arsenault B.J., Despres J.P., Vohl M.C. (2017). Precision Nutrition: A Review of Personalized Nutritional Approaches for the Prevention and Management of Metabolic Syndrome. Nutrients.

[22] Nizel A.E. (1972). Personalized nutrition counseling. ASDC J. Dent. Child..

[23] Brug J., Campbell M., van Assema P. (1999). The application and impact of computer-generated personalized nutrition education: A review of the literature. Patient Educ. Couns..

[24] Stewart-Knox B., Kuznesof S., Robinson J., Rankin A., Orr K., Duffy M., Poinhos R., de Almeida M.D., Macready A., Gallagher C. (2013). Factors influencing European consumer uptake of personalised nutrition. Results of a qualitative analysis. Appetite.

[25] Wang D.D., Hu F.B. (2018). Precision nutrition for prevention and management of type 2 diabetes. Lancet. Diabetes Endocrinol..

[26] Van Ommen B., van den Broek T., de Hoogh I., van Erk M., van Someren E., Rouhani-Rankouhi T., Anthony J.C., Hogenelst K., Pasman W., Boorsma A. (2017). Systems biology of personalized nutrition. Nutr. Rev..

[27] Daniel H., Klein U. (2016). Personalisierte Ernährung. J. Für Ernährungsmedizin.

[28] Nielsen D.E., El-Sohemy A. (2014). Disclosure of genetic information and change in dietary intake: A randomized controlled trial. PLoS ONE.

[29] Poinhos R., van der Lans I.A., Rankin A., Fischer A.R., Bunting B., Kuznesof S., Stewart-Knox B., Frewer L.J. (2014). Psychological determinants of consumer acceptance of personalised nutrition in 9 European countries. PLoS ONE.

[30] Janz N.K., Becker M.H. (1984). The Health Belief Model: A decade later. Health Educ. Q..

[31] Anderson A.S. (2000). How to implement dietary changes to prevent the development of metabolic syndrome. Br. J. Nutr..

[32] Bouchard C., Tremblay A. (1997). Genetic influences on the response of body fat and fat distribution to positive and negative energy balances in human identical twins. J. Nutr..

[33] Stunkard A.J., Sorensen T.I., Hanis C., Teasdale T.W., Chakraborty R., Schull W.J., Schulsinger F. (1986). An adoption study of human obesity. N. Engl. J. Med..

[34] Levy E., Menard D., Delvin E., Stan S., Mitchell G., Lambert M., Ziv E., Feoli-Fonseca J.C., Seidman E. (2001). The polymorphism at codon 54 of the FABP2 gene increases fat absorption in human intestinal explants. J. Biol. Chem..

[35] Hegele R.A., Harris S.B., Hanley A.J., Sadikian S., Connelly P.W., Zinman B. (1996). Genetic variation of intestinal fatty acid-binding protein associated with variation in body mass in aboriginal Canadians. J. Clin. Endocrinol. Metab..

[36] Tontonoz P., Hu E., Graves R.A., Budavari A.I., Spiegelman B.M. (1994). mPPAR gamma 2: Tissue-specific regulator of an adipocyte enhancer. Genes Dev..

[37] Tontonoz P., Hu E., Spiegelman B.M. (1994). Stimulation of adipogenesis in fibroblasts by PPAR gamma 2, a lipid-activated transcription factor. Cell.

[38] Deeb S.S., Fajas L., Nemoto M., Pihlajamaki J., Mykkanen L., Kuusisto J., Laakso M., Fujimoto W., Auwerx J. (1998). A Pro12Ala substitution in PPARgamma2 associated with decreased receptor activity, lower body mass index and improved insulin sensitivity. Nat. Genet..

[39] Hagg S., Ganna A., Van Der Laan S.W., Esko T., Pers T.H., Locke A.E., Berndt S.I., Justice A.E., Kahali B., Siemelink M.A. (2015). Gene-based meta-analysis of genome-wide association studies implicates new loci involved in obesity. Hum. Mol. Genet..

[40] Locke A.E., Kahali B., Berndt S.I., Justice A.E., Pers T.H., Day F.R., Powell C., Vedantam S., Buchkovich M.L., Yang J. (2015). Genetic studies of body mass index yield new insights for obesity biology. Nature.

[41] Thorleifsson G., Walters G.B., Gudbjartsson D.F., Steinthorsdottir V., Sulem P., Helgadottir A., Styrkarsdottir U., Gretarsdottir S., Thorlacius S., Jonsdottir I. (2009). Genome-wide association yields new sequence variants at seven loci that associate with measures of obesity. Nat. Genet..

[42] Speliotes E.K., Willer C.J., Berndt S.I., Monda K.L., Thorleifsson G., Jackson A.U., Lango Allen H., Lindgren C.M., Luan J., Magi R. (2010). Association analyses of 249,796 individuals reveal 18 new loci associated with body mass index. Nat. Genet..

[43] Loos R.J. (2018). The genetics of adiposity. Curr. Opin. Genet. Dev..

[44] Frayling T.M., Timpson N.J., Weedon M.N., Zeggini E., Freathy R.M., Lindgren C.M., Perry J.R., Elliott K.S., Lango H., Rayner N.W. (2007). A common variant in the FTO gene is associated with body mass index and predisposes to childhood and adult obesity. Science.

[45] Dina C., Meyre D., Gallina S., Durand E., Korner A., Jacobson P., Carlsson L.M., Kiess W., Vatin V., Lecoeur C. (2007). Variation in FTO contributes to childhood obesity and severe adult obesity. Nat. Genet..

[46] Scuteri A., Sanna S., Chen W.M., Uda M., Albai G., Strait J., Najjar S., Nagaraja R., Orru M., Usala G. (2007). Genome-wide association scan shows genetic variants in the FTO gene are associated with obesity-related traits. PLoS Genet..

[47] Claussnitzer M., Dankel S.N., Kim K.H., Quon G., Meuleman W., Haugen C., Glunk V., Sousa I.S., Beaudry J.L., Puviindran V. (2015). FTO Obesity Variant Circuitry and Adipocyte Browning in Humans. N. Engl. J. Med..

[48] Larder R., Sim M.F.M., Gulati P., Antrobus R., Tung Y.C.L., Rimmington D., Ayuso E., Polex-Wolf J., Lam B.Y.H., Dias C. (2017). Obesity-associated gene TMEM18 has a role in the central control of appetite and body weight regulation. Proc. Natl. Acad. Sci. USA.

[49] Wiemerslage L., Gohel P.A., Maestri G., Hilmarsson T.G., Mickael M., Fredriksson R., Williams M.J., Schioth H.B. (2016). The Drosophila ortholog of TMEM18 regulates insulin and glucagon-like signaling. J. Endocrinol..

[50] Cone R.D. (2005). Anatomy and regulation of the central melanocortin system. Nat. Neurosci..

[51] Farooqi I.S., Keogh J.M., Yeo G.S., Lank E.J., Cheetham T., O’Rahilly S. (2003). Clinical spectrum of obesity and mutations in the melanocortin 4 receptor gene. N. Engl. J. Med..

[52] Xiang L., Wu H., Pan A., Patel B., Xiang G., Qi L., Kaplan R.C., Hu F., Wylie-Rosett J., Qi Q. (2016). FTO genotype and weight loss in diet and lifestyle interventions: A systematic review and meta-analysis. Am. J. Clin. Nutr..

[53] Livingstone K.M., Celis-Morales C., Papandonatos G.D., Erar B., Florez J.C., Jablonski K.A., Razquin C., Marti A., Heianza Y., Huang T. (2016). FTO genotype and weight loss: Systematic review and meta-analysis of 9563 individual participant data from eight randomised controlled trials. BMJ.

[54] Sorensen T.I., Boutin P., Taylor M.A., Larsen L.H., Verdich C., Petersen L., Holst C., Echwald S.M., Dina C., Toubro S. (2006). Genetic polymorphisms and weight loss in obesity: A randomised trial of hypo-energetic high- versus low-fat diets. PLoS Clin. Trials.

[55] Papandonatos G.D., Pan Q., Pajewski N.M., Delahanty L.M., Peter I., Erar B., Ahmad S., Harden M., Chen L., Fontanillas P. (2015). Genetic Predisposition to Weight Loss and Regain with Lifestyle Intervention: Analyses From the Diabetes Prevention Program and the Look AHEAD Randomized Controlled Trials. Diabetes.

[56] Livingstone K.M., Celis-Morales C., Lara J., Ashor A.W., Lovegrove J.A., Martinez J.A., Saris W.H., Gibney M., Manios Y., Traczyk I. (2015). Associations between FTO genotype and total energy and macronutrient intake in adults: A systematic review and meta-analysis. Obes. Rev..

[57] Drabsch T., Gatzemeier J., Pfadenhauer L., Hauner H., Holzapfel C. (2018). Associations between Single Nucleotide Polymorphisms and Total Energy, Carbohydrate, and Fat Intakes: A Systematic Review. Adv. Nutr..

[58] Jiang L., Penney K.L., Giovannucci E., Kraft P., Wilson K.M. (2018). A genome-wide association study of energy intake and expenditure. PLoS ONE.

[59] Merino J., Dashti H.S., Li S.X., Sarnowski C., Justice A.E., Graff M., Papoutsakis C., Smith C.E., Dedoussis G.V., Lemaitre R.N. (2018). Genome-wide meta-analysis of macronutrient intake of 91,114 European ancestry participants from the cohorts for heart and aging research in genomic epidemiology consortium. Mol. Psychiatry.

[60] Larsen L.H., Angquist L., Vimaleswaran K.S., Hager J., Viguerie N., Loos R.J., Handjieva-Darlenska T., Jebb S.A., Kunesova M., Larsen T.M. (2012). Analyses of single nucleotide polymorphisms in selected nutrient-sensitive genes in weight-regain prevention: The DIOGENES study. Am. J. Clin. Nutr..

[61] Zhang X., Qi Q., Zhang C., Smith S.R., Hu F.B., Sacks F.M., Bray G.A., Qi L. (2012). FTO genotype and 2-year change in body composition and fat distribution in response to weight-loss diets: The POUNDS LOST Trial. Diabetes.

[62] Qi Q., Bray G.A., Smith S.R., Hu F.B., Sacks F.M., Qi L. (2011). Insulin receptor substrate 1 gene variation modifies insulin resistance response to weight-loss diets in a 2-year randomized trial: The Preventing Overweight Using Novel Dietary Strategies (POUNDS LOST) trial. Circulation.

[63] Celis-Morales C., Livingstone K.M., Marsaux C.F., Forster H., O’Donovan C.B., Woolhead C., Macready A.L., Fallaize R., Navas-Carretero S., San-Cristobal R. (2015). Design and baseline characteristics of the Food4Me study: A web-based randomised controlled trial of personalised nutrition in seven European countries. Genes Nutr..

[64] Saukko P. (2013). State of play in direct-to-consumer genetic testing for lifestyle-related diseases: Market, marketing content, user experiences and regulation. Proc. Nutr. Soc..

[65] Frankwich K.A., Egnatios J., Kenyon M.L., Rutledge T.R., Liao P.S., Gupta S., Herbst K.L., Zarrinpar A. (2015). Differences in Weight Loss Between Persons on Standard Balanced vs Nutrigenetic Diets in a Randomized Controlled Trial. Clin. Gastroenterol. Hepatol. Off. Clin. Pract. J. Am. Gastroenterol. Assoc..

[66] Steinberg G., Scott A., Honcz J., Spettell C., Pradhan S. (2015). Reducing Metabolic Syndrome Risk Using a Personalized Wellness Program. J. Occup. Environ. Med..

[67] Covolo L., Rubinelli S., Ceretti E., Gelatti U. (2015). Internet-Based Direct-to-Consumer Genetic Testing: A Systematic Review. J. Med. Internet Res..

[68] Reis A. (2011). Stellungnahme der Deutschen Gesellschaft fuer Humangenetik (GfH) zu “Direct-to-Consumer” (DTC)-Gentests. https://www.gfhev.de/de/leitlinien/LL_und_Stellungnahmen/2011_12_02_GfH-Stellungnahme_DTC-Gentests.pdf.

[69] Camp K.M., Trujillo E. (2014). Position of the Academy of Nutrition and Dietetics: Nutritional genomics. J. Acad. Nutr. Diet..

[70] Rafiq M., Ianuale C., Ricciardi W., Boccia S. (2015). Direct-to-consumer genetic testing: A systematic review of european guidelines, recommendations, and position statements. Genet. Test. Mol. Biomark..

[71] Bloss C.S., Ornowski L., Silver E., Cargill M., Vanier V., Schork N.J., Topol E.J. (2010). Consumer perceptions of direct-to-consumer personalized genomic risk assessments. Genet. Med. Off. J. Am. Coll. Med. Genet..

[72] Bray M.S., Loos R.J., McCaffery J.M., Ling C., Franks P.W., Weinstock G.M., Snyder M.P., Vassy J.L., Agurs-Collins T. (2016). NIH working group report-using genomic information to guide weight management: From universal to precision treatment. Obesity.

